# Chromosomal diversity and molecular divergence among three undescribed species of *Neacomys* (Rodentia, Sigmodontinae) separated by Amazonian rivers

**DOI:** 10.1371/journal.pone.0182218

**Published:** 2017-08-01

**Authors:** Willam Oliveira Da Silva, Julio Cesar Pieczarka, Malcolm Andrew Ferguson-Smith, Patricia Caroline Mary O’Brien, Ana Cristina Mendes-Oliveira, Iracilda Sampaio, Jeferson Carneiro, Cleusa Yoshiko Nagamachi

**Affiliations:** 1 Centro de Estudos Avançados da Biodiversidade, Laboratório de Citogenética, Instituto de Ciências Biológicas, Universidade Federal do Pará (UFPA), Belém, Brasil; 2 Cambridge Resource Centre for Comparative Genomics, Department of Veterinary Medicine, University of Cambridge, Cambridge, United Kingdom; 3 Laboratório de Zoologia e Ecologia de Vertebrados, ICB, Universidade Federal do Pará (UFPA), Pará, Brasil; 4 Laboratório de Genética e Biologia Molecular, Universidade Federal do Pará, Campus Universitário de Bragança, Pará, Brasil; National Cheng Kung University, TAIWAN

## Abstract

The *Neacomys* genus (Rodentia, Sigmodontinae) is distributed in the Amazon region, with some species limited to a single endemic area, while others may occur more widely. The number of species within the genus and their geographical boundaries are not known accurately, due to their high genetic diversity and difficulties in taxonomic identification. In this work we collected *Neacomys* specimens from both banks of the Tapajós River in eastern Amazon, and studied them using chromosome painting with whole chromosome probes of *Hylaeamys megacephalus* (HME; Rodentia, Sigmodontinae), and molecular analysis using haplotypes of mitochondrial genes COI and Cytb. Chromosome painting shows that *Neacomys* sp. A (NSP-A, 2n = 58/FN = 68) and *Neacomys* sp. B (NSP-B, 2n = 54/FN = 66) differ by 11 fusion/fission events, one translocation, four pericentric inversions and four heterochromatin amplification events. Using haplotypes of the concatenated mitochondrial genes COI and Cyt b, *Neacomys* sp. (2n = 58/FN = 64 and 70) shows a mean divergence of 6.2% for *Neacomys* sp. A and 9.1% for *Neacomys* sp. B, while *Neacomys* sp. A and *Neacomys* sp. B presents a medium nucleotide divergence of 7.4%. Comparisons were made with other published *Neacomys* data. The Tapajós and Xingu Rivers act as geographic barriers that define the distribution of these *Neacomys* species. Furthermore, our HME probes reveal four synapomorphies for the *Neacomys* genus (associations HME 20/[13,22]/4, 6a/21, [9,10]/7b/[9,10] and 12/[16,17]) and demonstrate ancestral traits of the Oryzomyini tribe (HME 8a and 8b, 18 and 25) and Sigmodontinae subfamily (HME 15 and 24), which can be used as taxonomic markers for these groups.

## Introduction

The Amazon is one of the richest biomes in terms of Brazilian mammalian species [1]. However, this biodiversity is not homogeneously distributed. Theories that consider ecologic, morphologic, chromosomal and/or molecular analysis performed with terrestrial vertebrate taxonomic groups have shown the occurrence of distinct biogeographic regions in the Amazon [2–4].

The large Amazonian rivers are suggested as major geographic barriers to species distribution in the region [3, 5, 6]. The Riverine Barrier Hypothesis was first proposed by Wallace [7] and reviewed by many authors [8, 9]. Silva et al. [5] recognize eight distinct endemic areas for Amazonian species limited by the major rivers: Belém, Guiana, Imeri, Inambari, Napo, Rondônia, Tapajós and Xingu. Each one of them has a distinct evolutionary history, with regard to species diversification.

Taxonomic studies in the Amazonian regions have been difficult because of overlapping characteristics between distinct species [10]. The taxonomy issue of rodents from the Sigmodontinae subfamily (Rodentia, Cricetidae) has been a problem, since this subfamily belongs to one of the most complex and diverse group of New World mammals [11, 12].

Recently, genetic strategies have helped to solve problems related to evolution and taxonomy, such as the comparative analysis of mitochondrial gene sequences of Cytochrome C Oxidase—subunit I (COI) and Cytochrome b (Cytb), frequently used for the comparison of species in the same genus or the same family [13]. Also, chromosome painting has been useful for karyotypic evolution studies based on cross-species chromosome homology [14, 15], but only 24 species from seven genera among the Sigmodontinae have been analyzed by this technique [16–23]. *Neacomys* has been one genus in which the understanding of karyotype evolution is complicated by the number of species, geographic boundaries and phylogenetic relationships.

The *Neacomys* genus Thomas, 1900 (Sigmodontinae, Oryzomyini) currently includes eight valid species with a known distribution in Central and South America, and only two species that do not occur in the Brazilian Amazon (*N*. *pictus and N*. *tenuipes*) [2, 24–28] (Table 1). However, the occurrence and geographic boundaries of the distribution of *Neacomys* species are poorly known, as are a large number of Amazon terrestrial mammalian taxa [2, 29].

**Table 1:** Cytogenetic data available for *Neacomys* genus, with diploid number (2n) and autosomal fundamental number (FN).

Species	2n	FN	References
*N*. *dubosti* Voss, Lunde, and Simmons, 2001	62	-	Voss et al. [25]
*N*. *dubosti* Voss, Lunde, and Simmons, 2001	64	68	Da Silva et al. [28]
*N*. *guianae* Thomas, 1905	56	-	Baker et al. [24]
*N*. *minutus* Patton, da Silva, and Malcolm, 2000	35–36	40	Patton et al. [29]
*N*. *musseri* Patton, da Silva, and Malcolm, 2000	34	64–68	Patton et al. [29]
*N*. *paracou* Voss, Lunde, and Simmons, 2001	56	-	Voss et al. [25]
*N*. *paracou* Voss, Lunde, and Simmons, 2001	56	62, 66	Da Silva et al. [28]
*Neacomys* sp.	58	64, 70	Da Silva et al. [28]
*N*. *spinosus* Thomas, 1882	64	68	Patton et al. [29]
*N*. *tenuipes* Thomas, 1900	56	-	Pérez-Zapata et al. (1996) *apud* Redi et al. [26]

Cytogenetic studies of the genus *Neacomys* reveal variation in the diploid number from 34 to 64 and the fundamental number (FN) from 40 to 70 (Table 1). Recently Da Silva et al. [28] studied three *Neacomys* species and described five new karyotypes: one for *N*. *dubosti* (2n = 64/FN = 68), two for *N*. *paracou* (2n = 56/FN = 62 and 66) and two for *Neacomys* sp. (2n = 58/FN = 64 and 70). Furthermore, the authors also generated a molecular phylogeny using Cytb, confirming the monophyly of *Neacomys* [2, 25, 30–32] and the status of *Neacomys* sp. as a previously undescribed species.

In order to test the Riverine Barrier Hypothesis, the present study compared *Neacomys* from different banks of Amazon rivers. We defined the karyotypes of two undescribed species of *Neacomys*, *Neacomys* sp. A (NSP-A, 2n = 58/FN = 68) and *Neacomys* sp. B (NSP-B, 2n = 54/FN = 66), collected from the right and left banks respectively, of the Tapajós River in Eastern Amazon. Whole chromosome probes of *Hylaeamys megacephalus* (HME) [20], were used to determine regions of chromosomal homology, and the mitochondrial genes COI and Cytb were used for molecular analysis. These results were compared with those from an undescribed species mentioned by da Silva et al. [28]. We present biogeographic inferences and discuss the chromosomal evolution of these taxa.

## Material and methods

Animals collected during this study were handled following procedures recommended by the American Society of Mammalogists. JCP has a permanent field permit, number 13248 from “Instituto Chico Mendes de Conservação da Biodiversidade”. The Cytogenetics Laboratory from UFPa has permit number 19/2003 from the Ministry of Environment for sample transport and permit 52/2003 for using the samples for research. The Ethics Committee (Comitê de Ética Animal da Universidade Federal do Pará) approved this research (Permit 68/2015). The rodents were maintained in the lab with food and water, free from stress, until their euthanasia using intraperitoneal injection of barbiturate (Pentobarbital, 120 mg/kg) after local anesthetic (lidocaine used topically).

**Fig 1:**
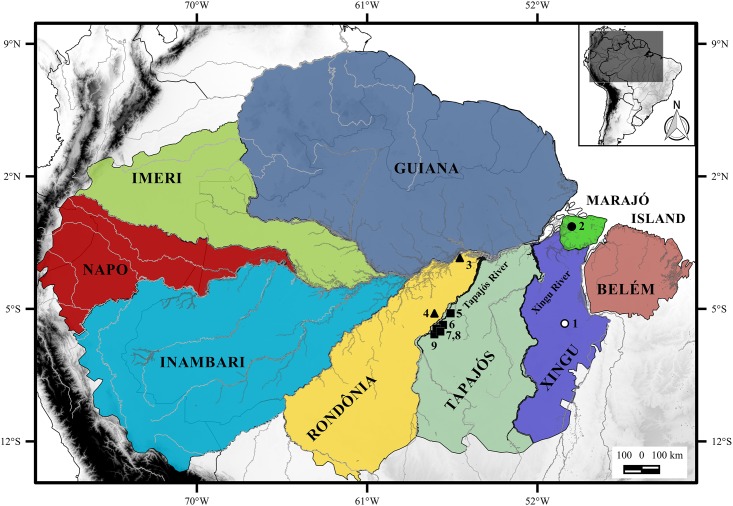
Amazon endemic areas based on the distribution of terrestrial vertebrates [5]. Collection points of *Neacomys* sp. A (NSP-A; Black square), *Neacomys* sp. B (NSP-B; black triangle) and *Neacomys* sp. (white and black circle) [28]. Tapajós and Xingu Rivers are highlighted in black. The numbers refer to localities mentioned in S1 Table. (1) Marabá; (2) Chaves, Marajó island; (3) Juruti; (4, 5, 6 and 8) Itaituba; (7 and 9) Jacareacanga, all in Pará state, Brazil.

We studied the karyotypes of nine specimens (five males and four females including one fetus) of *Neacomys* sp. A, collected from the right bank of the Tapajós River, in the Itaituba (Fig 1, localities 5, 6 and 8) and Jacareacanga municipalities (Fig 1, localities 7 and 9), Pará state, Brazil; seven specimens of *Neacomys* sp. B, three (one female and two males—one fetus) from the Itaituba municipality (Fig 1, locality 4) and four (three males and one female) from the Juruti municipality (Fig 1, locality 3), both from the left bank of the Tapajós River, Pará state, Brazil (S1 Table).

Samples were collected using Pitfall traps [33] and deposited at the mammal collection of Museu de Zoologia da Universidade Federal do Pará (UFPA), in Belém, Pará, Brazil. Chromosomal preparations were obtained from bone marrow [34] and by fibroblast cell culture made from two fetuses, established in the Centro de Estudos Avançados da Biodiversidade, Laboratório de Citogenética, Instituto de Ciências Biológicas, Universidade Federal do Pará, Belém, Pará, Brazil. G-banding and C-banding were performed following Sumner et al. [35] and Sumner [36], respectively. Fluorescent *in situ* Hybridization (FISH) studies were made using telomeric probes (All Telomere, ONCOR) and chromosome painting with whole chromosome probes of HME [20]. Twenty-one of the 24 probes of HME correspond to one HME chromosome pair, while three probes correspond to two pairs ([9,10]; [13,22]; [16,17]). Digital images were obtained by Nis-Elements software and Nikon H550S microscopy. Chromosome classification followed Levan et al. [37].

The map was made using QUANTUM-GIS (QGIS) program version 2.10.1. Database were obtained from DIVA and IBGE.

We used sequences of 625 base pairs from 26 samples for Cytochrome C Oxidase—subunit I (COI), with 20 new sequences (*Neacomys* sp. A and *Neacomys* sp. B) and six sequences obtained from GenBank (S1 Table). For Cytochrome b (Cytb) we used sequences of 801 base pairs from 32 samples, with 18 new (*Neacomys* sp. A and *Neacomys* sp. B), and the others were kindly supplied by J.L. Patton (Museum of Vertebrate Zoology, Berkeley) or retrieved from GenBank (S1 Table). We included data from da Silva et al. [28] on an undescribed species (here mentioned as “*Neacomys* sp.”) found in Marabá and Marajó Island (Fig 1, places 1 and 2).

The DNA was extracted with Wizard^®^ Genomic DNA Purification Kit (Promega, Madison, WI, USA). COI gene fragment amplification was made with the primers Fish F1 [5’- TCAACCAACCACAAAGACATTGGC AC-3’] and Fish R1 [5’-TAGACTTCTGGGTGGCCAAAGA ATCA-3’] [38], and Cytb was made with the primers MVZ-05 CGAAGCTTGATATGAAAAACCATCGTTG [38] and MVZ-16 AAATAGGAARTATCAYTCTGGTTTRAT [39].

The DNA sequencing used the Big Dye ABI PRISMTM Dye Terminator Cycle Sequencing kit, in the automated sequencer ABI 3500 (Applied Biosystems—Carlsbad, CA, USA).

The two markers were concatenated and phylogenetic analyzes were performed from haplotypes. The evolutionary model was generated by the software Kakusan v. 4–4.0.2015.01.23 [40], which selected GTR + GAMA as the most appropriate evolutionary model.

The genetic distance between taxa was estimated with Molecular Evolutionary Genetics Analysis—MEGA v. 6.0 software [41], recovering K2P model.

The phylogenetic reconstructions were made using both the maximum likelihood (ML) method, run in RaxML v. 8 [42] with 1000 bootstrap replicates and Bayesian inference (BI) as implemented in MrBayes v. 3.2.1 [43]. In MrBayes, the analysis of substitution model parameters was unlinked across partitions. Two independent runs were initiated simultaneously with four independent Markov-Chain Monte Carlo (MCMC) chains (one cold and three heated). The MCMC algorithm was based on 700,000 cycles (generations), sampled every 5,000 cycles, with 20% of the samples being discarded as burn-in. Convergence was assessed by comparing the two runs. The MCMC output was visualized and diagnosed in Tracer v. 1.6 [44]. The run was considered satisfactory when, for all traces, the Effective Sample Size (ESS) values were over 200. *Hylaeamys megacephalus*, *Oecomys rutilus*, *O*. *concolor*, *Deltamys* and *Thalpomys* were used as outgroup. All these species belong to the Sigmodontinae subfamily and are phylogenetically close to *Neacomys*, according to Weksler [30].

Divergence time estimates were performed using BEAST 1.8.3 [45]. For calibration, we use three calibration points (4.4 Ma corresponding to separation time estimate between *Oecomys* and *Hylaeamys* [46]; 4.5 Ma corresponding to separation between *Neacomys* and *Thalpomy*s; and 5 Ma corresponding to separation between *Deltamys* and the *Neacomys*/*Thalpomys* clade). Uncorrelated relaxed clock was assigned to the length rates among branches and Yule prior was used for the tree. Four independent runs were made of 20^5^ generations, showing parameters and trees every 2,500 generations. The convergence of races was evaluated in Tracer v. 1.6 [44], assuming ESS values above 200 as satisfactory. Tree’s and log file’s results were summarized in TreeAnnotator v. 1.8.3 and LogCombiner v. 1.8.3 [47], respectively; we discard 20% as burn-in. The tree was displayed and edited in Figtree v. 1.4.2 (http://tree.bio.ed.ac.uk/software/figtree/).

Estimates of ancestral areas were generated by Vicariance-Dispersion analysis (S-DIVA) implemented in RASP v. 3.2 [48]. The terminal taxa were coded correlating their ranges to areas of endemism of the Amazon (Belém, Xingu, Tapajós, Rondônia, Inambari, Guiana, Imeri and Napo) and Marajó Island [5]. The maximum number of ancestral areas chosen was three.

## Results

### Classic cytogenetics

**Fig 2:**
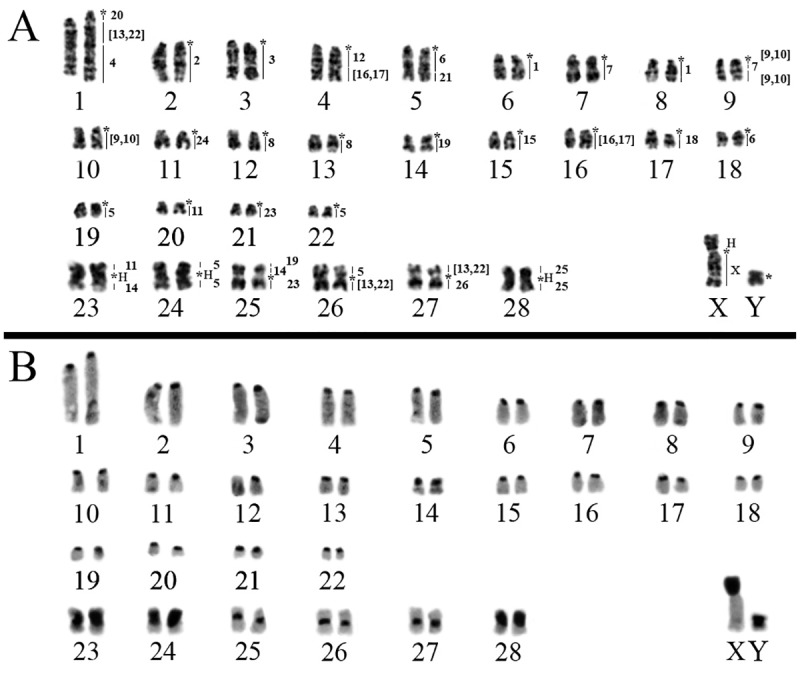
*Neacomys* sp. A (2n = 58/FN = 68). A) G-banding with chromosome painting with HME probes. B) C-banding (sequential). (H) Large block of constitutive heterochromatin. (*) Indicates centromere.

*Neacomys* sp. A (NSP-A; Fig 1, localities 5–9) have 2n = 58/FN = 68 (Fig 2A) with autosomes comprising 22 acrocentric pairs (1–22) and six meta/submetacentric pairs (23–28); the X chromosome is a middle-sized submetacentric and the Y chromosome is a small-sized submetacentric. Constitutive Heterochromatin (CH) is distributed at the centromeric region of almost all autosomes. Pairs 23, 24 and 28 present large blocks of CH at a pericentromeric region. The X chromosome has a large CH block in the short arm, and the Y chromosome is almost entirely heterochromatic (Fig 2B).

**Fig 3:**
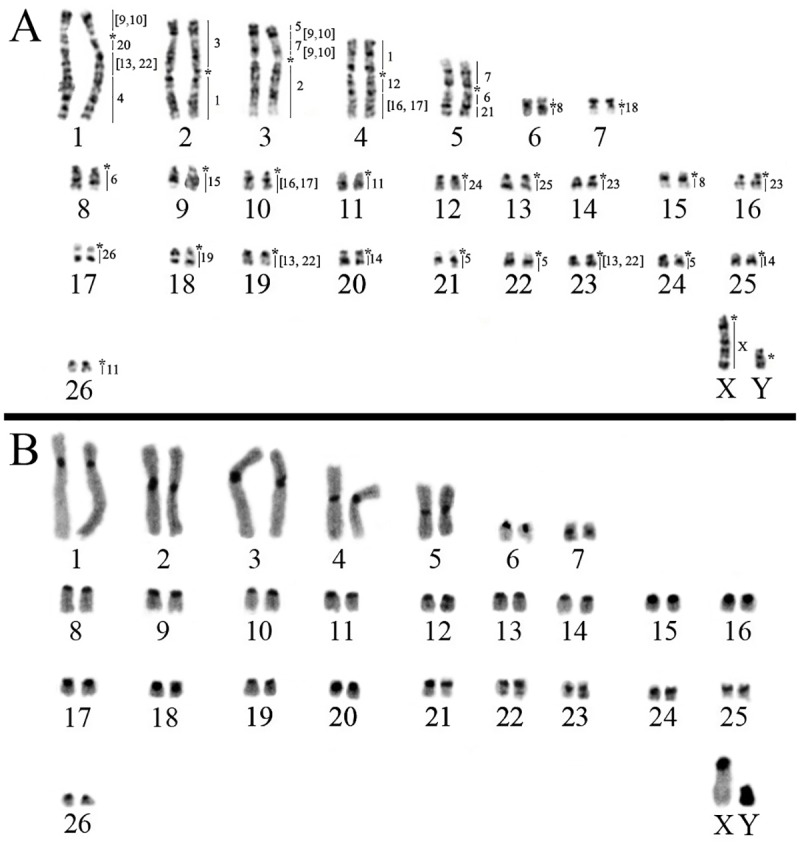
*Neacomys* sp. B (2n = 54/FN = 66). A) G-banding with chromosome painting with HME probes. B) C-banding. (*) Indicates centromere.

*Neacomys* sp. B (NSP-B; Fig 1, localities 3 and 4) have 2n = 54/FN = 66 (Fig 3A) with seven meta/submetacentric autosomes pairs (1–7) and 19 acrocentric pairs (8–26). The X chromosome is a middle-sized acrocentric and the Y chromosome is small-sized. CH is distributed along the centromeric region of all autosomes and the X chromosome; the Y chromosome is almost entirely heterochromatic (Fig 3B).

### Molecular cytogenetics

The FISH results of 24 HME whole chromosome probes [20] on two *Neacomys* species are detailed in Table 2 and Figs 2A and 3A. Centromeric (*) and heterochromatic (H) regions do not show hybridization signals. The hybridization of each HME probe on two *Neacomys* species is detailed in S1 Fig.

**Table 2:** Chromosomal homology among *Hylaeamys megacephalus* (HME), *Cerradomys langguthi* (CLA) [20], *Thaptomys nigrita* (TNI), *Akodon montensis* (AMO) [23], *Akodon* sp. (ASP), *Necromys lasiurus* (NLA) [22], *Neacomys* sp. A (NSP-A) and *Neacomys* sp. B (NSP-B).

HME 2n = 54 FN = 62	TNI 2n = 52 FN = 52	AMO 2n = 24 FN = 42	ASP 2n = 10 FN = 14	NLA 2n = 34 FN = 34	CLA 2n = 46 FN = 62	NSP-A 2n = 58 FN = 68	NSP-B 2n = 54 FN = 66
**1**	4, 8	1q distal, 4q	1q interstitial, 2q distal	5q distal, 7	2q, 20	6, 8	2p, 4q
**2**	7, 12	1q interstitial, 7q	1p distal, 2q interstitial	9q interstitial, 13	10, 18, 19	2	3q
**3**	1 interstitial and distal	2q	2p distal	3q distal	1q interstitial, 3p	3	2p
**4**	13, 15	1p proximal, 5p distal	2q proximal and interstitial, 3q interstitial	1q proximal, 10q proximal, 11q distal	5, 13	1q distal	1q distal
**5**	2 distal, 5 proximal, 6 proximal	3q interstitial, 6p interstitial, 10	1p interstitial and proximal, 3q interstitial (2 segments)	6q distal, 12q interstitial, 14q distal	1p distal, 1q proximal, 8	19, 22, 24, 26p	3p distal, 21, 22, 24
**6**	3 proximal and interstitial	2p	2p interstitial	2q interstitial	4q distal	5q proximal, 18	5q proximal, 8
**7**	18	5q proximal, 8q	1p interstitial, 3q interstitial	1q interstitial, 4q proximal	3q interstitial	7, 9q interstitial	3p interstitial, 5p
**8**	6 distal	3p	3q proximal	1qinterstitial	4q proximal, 7	12, 13	6, 15
**[9,10]**	2 proximal, 5 distal	5q, 9p	1q interstitial, 3q interstitial	1q distal, 6q proximal	2p distal, 3q distal	9q (two different segments), 10	1p, 3p interstitial (two different segments)
**11**	9 distal, 10 proximal	1p interstitial, 6q distal	2q interstitial, 3q distal	10q interstitial, 12q distal	11q proximal, 6	20, 23p	11, 26
**12**	16	1q interstitial	2q interstitial	5q proximal	2p proximal	4q proximal	4q proximal
**[13,22]**	9 proximal, 11 interstitial and distal, 21	3q proximal, 4p distal, 6q proximal	1q interstitial, 3q interstitial (two segments)	4q distal, 12q proximal, 14q proximal	1q (two different segments), 9	1q interstitial, 26q, 27p	1q interstitial, 19, 23
**14**	17 proximal, 24	6p proximal, 8p interstitial	1p interstitial, 3q interstitial	2q interstitial, 15q interstitial	1p interstitial, 21	23q, 25p proximal	20, 25
**15**	19	9q	1q distal	8q distal	12	15	9
**[16,17]**	10 distal, 22	1p distal, 3q distal	2p proximal, 2q interstitial, 3qinterstitial	2q proximal, 10q distal	1q proximal, 11q distal	4q distal, 16	4q distal, 10
**18**	1 proximal, 23	1q proximal, 5p proximal	2pinterstitial, 2q interstitial, 3q interstitial	3q proximal, 9q proximal	16	17	7
**19**	17 distal	8p distal, 8q proximal	1p interstitial (two segments)	15q proximal and distal	1p interstitial, 3q proximal	14, 25p distal	18
**20**	11 proximal	4q proximal	1q proximal and interstitial	4q interstitial	1q distal	1q proximal	1q proximal
**21**	3 distal	2p distal	2p interstitial	2q distal	4p, 4q interstitial	5q distal	5q distal
**23**	20	7p	1p interstitial	8q proximal	15	21, 25q	14, 16
**24**	14	6p distal	3q interstitial (two segments)	9q distal	14	11	12
**25**	1 proximal	2p proximal	2p interstitial, 3q interstitial	3q interstitial, 11q proximal	17	28	13
**26**	25	11	4	16	22	27q	17
**X**	X	X (Xq)	X	X	X	Xq	X
**Total**	36 signs	38 signs	45 signs	40 signs	40 signs	40 signs	39 signs

#### *Neacomys* sp. A (NSP-A, 2n = 58 and FN = 68)

FISH with HME probes shows 40 hybridization signals in NSP-A (Fig 2A, Table 2). Eleven autosomes plus the X chromosome show conserved synteny. From those eleven, six (HME 2, 3, 15, 18, 24 and 25) hybridize whole chromosomes of NSP-A (2, 3, 15, 17, 11 and 28, respectively) and five (HME 4, 12, 20, 21 and 26) are associated with regions of other chromosomes (1q distal, 4q proximal, 1q proximal, 5q distal and 27q, respectively).

The other twelve autosomal probes show multiple signals in NSP-A, with ten (HME 1, 6, 7, 8, [9,10], 11, 14, [16,17], 19 and 23) hybridizing to two chromosomes each, where HME 1 and 8 hybridize to two whole distinct chromosomes each while the others hybridize to a chromosome and a portion of another chromosome. HME [13,22] show signals in three chromosomes and HME 5 in four different chromosomes.

**Fig 4:**
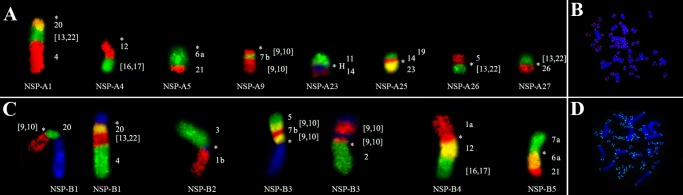
A) Chromosomal associations of *Neacomys* sp. A 1, 4, 5, 9, 23, 25, 26 and 27; B) FISH with telomeric probes in *Neacomys* sp. A. C) Chromosomal associations of *Neacomys* sp. B 1, 2, 3, 4 and 5; D) FISH with telomeric probes in *Neacomys* sp. B. (H) Indicates large block of constitutive heterochromatin. (*) Indicates centromere.

Eight NSP-A pairs show chromosomal associations (to multiple HME probes): pair 1 (HME */20/[13,22]/4), pair 4 (HME */12/[16,17]), pair 5 (HME */6a/21), pair 9 (HME */[9,10]/7b/[9,10]), pair 23 (11/*/14), pair 25 (19/14/*/23), pair 26 (5/*/[13,22]) and pair 27 ([13,22]/*/26) (Table 2; Figs 2A and 4A). FISH with telomeric probes show signals only at the distal ends of chromosomes (Fig 4B).

#### *Neacomys* sp. B (NSP-B, 2n = 54 and FN = 66)

FISH with HME probes show 39 hybridization signals in NSP-B (Fig 3A, Table 2). Twelve autosomes plus the X chromosome show conserved synteny. From those twelve, six (HME 15, 18, 19, 24, 25 and 26) hybridized to whole chromosomes of NSP-B (9, 7, 18, 12, 13 and 17, respectively) and six (2, 3, 4, 12, 20 and 21) are associated with regions of other chromosomes (NSP-B 3q, 2p, 1q distal, 4q proximal, 1q proximal and 5q distal, respectively).

The other eleven autosomal probes show multiple signals in NSP-B, with nine (HME 1, 6, 7, 8, [9,10], 11, 14, [16,17] and 23) hybridizing to two chromosomes each; HME [13,22] show signals in three chromosomes and HME 5 in four different chromosomes.

Five NSP-B pairs present chromosomal associations: pair 1 (HME [9,10]/*/20/[13,22]/4), pair 2 (HME 3/*/1b), pair 3 (HME 5/[9,10]/7b/[9,10]/*/2), pair 4 (HME 1a/*/12/[16,17]) and pair 5 (7a/*/6a/21) (Table 2; Figs 3A and 4C). FISH with telomeric probes show signals only at the distal ends of chromosomes (Fig 4D).

### Molecular phylogeny

**Fig 5:**
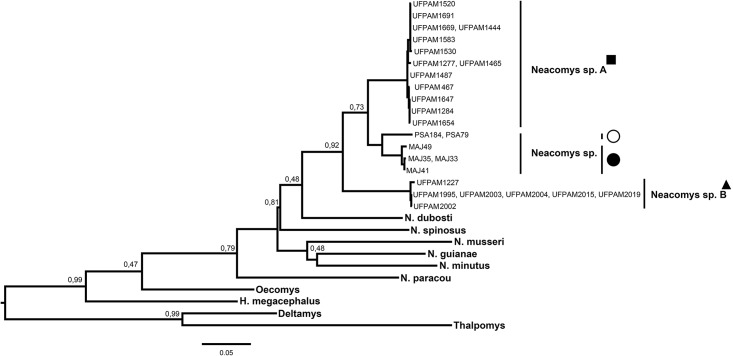
Maximum likelihood tree of specimens of *Neacomys*, based on haplotypes from 58 sequences of the concatenated mitochondrial genes (COI and Cytb). Bootstrap values are shown above the nodes. The symbols refer to species mentioned in Fig 1. Legend: black square (*Neacomys* sp. A), black triangle (*Neacomys* sp. B), white circle (*Neacomys* sp. from Marabá) and black circle (*Neacomys* sp. from Marajó island) [28].

**Fig 6:**
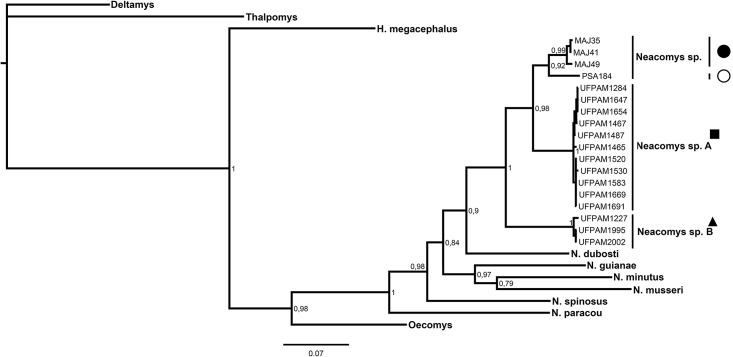
Bayesian inference chronogram from BEAST estimated based on haplotypes from 58 sequences of the concatenated mitochondrial genes (COI and Cytb). The Bayesian posterior probability (BPP) is given at each node (BS/BPP). The symbols refer to species mentioned in Fig 1. Legend: black square (*Neacomys* sp. A), black triangle (*Neacomys* sp. B), white circle (*Neacomys* sp. from Marabá) and black circle (*Neacomys* sp. from Marajó island) [28].

The genus *Neacomys* was shown to be monophyletic in both analysis of maximum likelihood and Bayesian inference (Figs 5 and 6, respectively) for the concatenated mitochondrial genes (COI and Cytb; Table 3), supported by maximum values of bootstrap posterior probability. Six valid species were recovered for the clades: *N*. *dubosti*, *N*. *guianae*, *N*. *minutus*, *N*. *musseri*, *N*. *paracou* and *N*. *spinosus*. Besides, our data recovered two new clades (*Neacomys* sp. A and *Neacomys* sp. B), being monophyletic, with a high degree of divergence between them (Table 3), and other species in the *Neacomys* genus. NSP-A and NSP-B clades are described for the first time in this study.

**Table 3:** Mean genetic distances of the concatenated mitochondrial genes Cytochrome C Oxidase—Subunit I (COI) and Cytochrome b (Cytb) according to Kimura-2 parameters among different *Neacomys* species recovered in the present study. Values are in percentage (%).

Species	1	2	3	4	5	6	7	8	9	10	11
[1] *Neacomys* sp.											
[2] *Neacomys* sp. A	6,2										
[3] *Neacomys* sp. B	9,1	7,4									
[4] *N*. *guianae*	15,8	14,4	15,6								
[5] *N*. *spinosus*	13,7	13,5	13,0	15,7							
[6] *N*. *dubosti*	10,6	10,8	11,2	14,6	14,2						
[7] *N*. *paracou*	15,4	15,6	16,4	16,6	15,5	16,3					
[8] *N*. *musseri*	15,1	15,7	15,1	14,9	15,8	15,6	16,9				
[9] *N*. *minutus*	12,9	13,4	14,0	12,9	16,6	16,1	16,5	14,1			
[10] *Oecomys*	15,4	16,6	16,9	17,9	16,1	13,7	13,5	16,3	18,1		
[11] *H*. *megacephalus*	18,5	19,0	17,9	17,7	19,6	17,5	17,3	20,3	19,8	14,9	

*Neacomys* sp. samples from Marabá and Marajó Island (Fig 1, localities 1 and 2, respectively) [28] and *Neacomys* sp. A are sister lineages (Figs 5 and 6), but phylogenetically distinct. The average nucleotide divergence between *Neacomys* sp. A and *Neacomys* sp. is 6.2%, while between *Neacomys* sp. B and *Neacomys* sp. is about 9.1%, and *Neacomys* sp. A and *Neacomys* sp. B is about 7.4%, both distance estimates for the concatenated mitochondrial genes (COI and Cytb; Table 3).

### Divergence time estimates and ancestral areas

**Fig 7:**
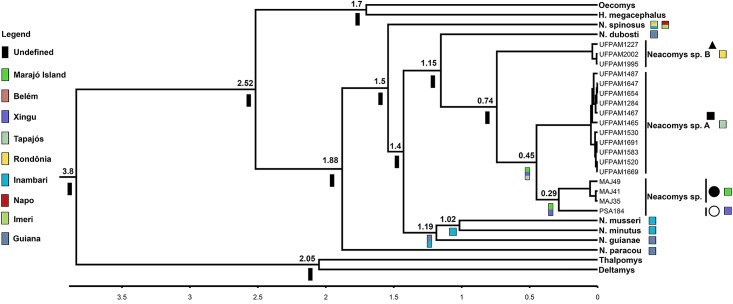
Chronogram derived from a Bayesian analysis of the concatenated mitochondrial genes (COI and Cytb) of *Neacomys* genus. The scale shows divergence times as millions of years ago (Ma). Colored bars correspond to ancestral areas recovered by Vicariance-Dispersion analysis, to Marajó island and Amazon endemic areas mentioned in Fig 1. The symbols refer to species mentioned in Fig 1. Legend: black square (*Neacomys* sp. A), black triangle (*Neacomys* sp. B), white circle (*Neacomys* sp. from Marabá) and black circle (*Neacomys* sp. from Marajó island) [28].

Our divergence time estimates suggest that the diversification of species currently recognized for *Neacomys* genus occurred in the last 1.88 Ma. The last split was between *Neacomys* sp. and *Neacomys* sp. A about 0.45 Ma (Fig 7).

Our data were unable to recover some *Neacomys* ancestor nodes with high statistical support values. However, they recovered with maximum support the ancestor of *N*. *guianae*, *N*. *musseri* and *N*. *minutus* that occurred in current areas of Inambari and Guiana. Thus, the ancestor of *Neacomys* sp. was endemic in the Marajó Island and Xingu area.

## Discussion

### Karyotypic and phylogenetic analyses of *Neacomys*

*Neacomys* sp. A (2n = 58/FN = 68; Fig 1, localities 5–9) presents a similar karyotype to those described by Da Silva et al. [28] for an undescribed species, identified as *Neacomys* sp., for which two karyotypes are described: 2n = 58/FN = 64 for Marabá, in the southeastern portion of the state of Pará (Fig 1, locality 1) and 2n = 58/FN = 70 for specimens from Marajó Island (Fig 1, locality 2). Comparative analysis by G- and C-banding demonstrate that the differences in FN among the three karyotypes are due to differences in heterochromatic blocks, in which CH forms the short arms of some bi-armed chromosomes.

*Neacomys* sp. B (2n = 54/FN = 66) presents a new karyotype for the genus when compared with species with 2n between 56 and 64 (*N*. *dubosti*, *N*. *guianae*, *N*. *paracou*, *Neacomys* sp. and *N*. *spinosus*, Table 1). In NSP-B the bigger autosomes pairs are metacentric and submetacentric, while in other species they are medium-size acrocentrics, indicating multiple fusion/fission and/or translocation events.

According to Bradley and Baker [49] and Baker and Bradley [50], who made a meta-analysis of the genetic divergence in the Cytb gene for many groups of rodents, values of genetic divergence below 2% were present in different populations of the same species; over 5% are associated with potentially unrecognized species, and over 10% belongs to different species.

Recently, genetic approaches among eight *Neacomys* species performed by Da Silva et al. [28] found genetic divergences ranging from 10–21% (Cytb). Our results shows values ranging from 6.2–20.3% (Table 3), both are in agreement with interspecies variation values for rodents [49, 50]. Moreover, *Neacomys* sp. populations [28] from Marabá and Marajó Island (Fig 1, localities 1 and 2, respectively) constitute a single species, with an average intraspecific genetic divergence of 2% (see above).

However, *Neacomys* sp. shows a mean divergence of 6.2% for *Neacomys* sp. A and 9.1% for *Neacomys* sp. B, while *Neacomys* sp. A and *Neacomys* sp. B present a medium nucleotide divergence of 7.4% from each other in concatenated mitochondrial genes (COI and Cyt b; Table 3). These three taxa present >10% of divergence from other *Neacomys* species in both analyses.

Thus, based on the genetic species concept [49, 50] and the karyotypic and molecular data of this study, we conclude that *Neacomys* sp. A and *Neacomys* sp. B are two undescribed species within the genus and distinct from the undescribed species (*Neacomys* sp.) proposed by Da Silva et al. [28]. Moreover, these three undescribed species may represent cryptic species, which reinforces a taxonomic analysis to define their taxonomic status.

### Chromosomal rearrangements and signatures

**Fig 8:**
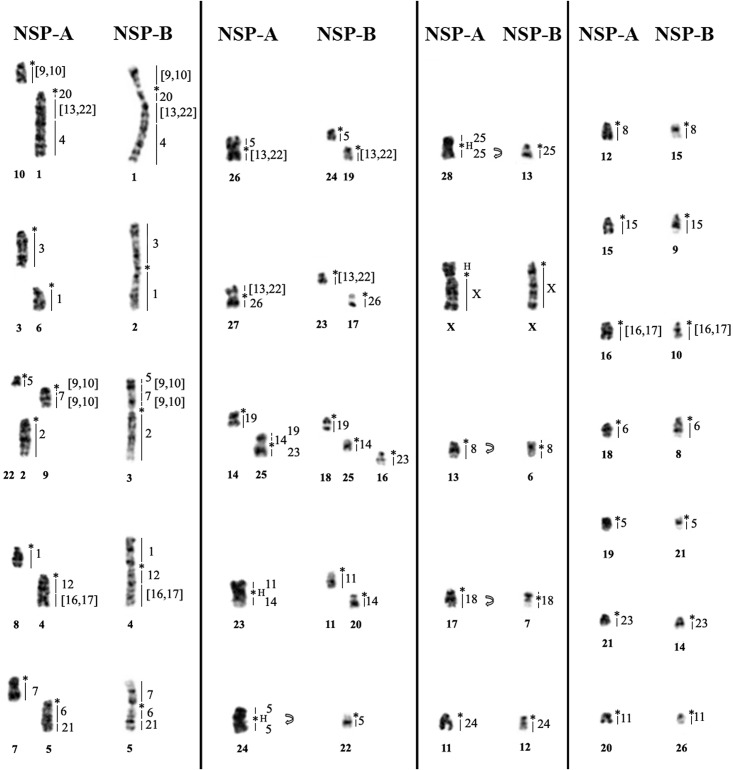
Comparative analysis by G-banding and ZOO-FISH with HME whole chromosome probes [20], between *Neacomys* sp. A and *Neacomys* sp. B. (H) Large block of constitutive heterochromatin. (*) Indicates centromere. Curved arrow indicates pericentric inversion.

The comparison of *Neacomys* sp. A and *Neacomys* sp. B karyotypes show that these species had a karyotypic evolutionary history that involved complex rearrangements with some chromosomal signatures that differ them from other Sigmodontinae (see below), as also many autapomorphic characteristics for each species which confirm that this genus is very diverse even in karyotypes with not very distant 2n, and they differ from one another by 11 fusion/fission events and one translocation in 16 pairs of NSP-A and 14 pairs of NSP-B, plus four pericentric inversions in four autosomal pairs, and four CH amplification events in three autosomal pairs and the X chromosome. Only eight chromosomal pairs show conserved synteny with no detectable change (Table 4, Fig 8).

**Table 4:** NSP-A and NSP-B rearrangements involved.

Rearrangement	NSP-A (2N = 58/FN = 68)	NSP-B (2N = 54/FN = 66)
Fusion/Fission	10 (*HME [9,10]) 1 (*HME 20/[13,22]/4)	1p (HME [9,10]*20/[13,22]/4) 1q (HME [9,10]*20/[13,22]/4)
Fusion/Fission	3 (*HME 3) 6 (*HME 1)	2p (HME 3*1) 2q (HME 3*1)
Fusion/Fission	22 (*HME 5) 9 (*HME [9,10]/7/[9,10]) 2 (*HME 2)	3p distal (HME 5/[9,10]/7/[9,10]*2) 3p prox+inters. (HME 5/[9,10]/7/[9,10]*2) 3q (HME 5/[9,10]/7/[9,10]*2)
Fusion/Fission	8 (*HME 1) 4 (*HME 12/[16,17])	4p (HME 1*12/[16,17]) 4q (HME 1*12/[16,17])
Fusion/Fission	7 (*HME 7) 5 (*HME 6/21)	5p (HME 7*6/21) 5q (HME 7*6/21)
Fusion/Fission	26 (HME 5*[13,22])	24 (*HME 5) + 19 (*HME [13,22])
Fusion/Fission	27 (HME [13,22]*26)	23 (HME *[13,22]) + 17 (*HME 26)
Fusion/Fission	25 (HME 19/14*23)	18qdist (*HME 19)+ 25 (*HME 14)+ 16 (*HME 23)
Translocation	14 (*HME 19)+ 25pdistal (HME 19/14*23)	18 (*HME 19)
Fusion/Fission+ H Amplification /Deletion	23 (HME 11H*H14)	11 (*HME 11) + 20 (*HME 14)
Pericentric Inversion+ H Amplification /Deletion	24 (HME 5H*H5)	22 (*HME 5)
Pericentric Inversion+ H Amplification /Deletion	28 (HME 25H*H25)	13 (*HME 25)
H Amplification /Deletion	X (H*HME X)	X (*HME X)
Pericentric Inversion	13 (*HME 8)	6 (HME 8*8)
Pericentric Inversion	17 (*HME 18)	7 (HME 18*18)
Conserved	11 (*HME 24)	12 (*HME 24)
Conserved	12 (*HME 8)	15 (*HME 8)
Conserved	15 (*HME 15)	9 (*HME 15)
Conserved	16 (*HME [16,17])	10 (*HME [16,17])
Conserved	18 (*HME 6)	8 (*HME 6)
Conserved	19 (*HME 5)	21 (*HME 5)
Conserved	21 (*HME 23)	14 (*HME 23)
Conserved	20 (*HME 11)	26 (*HME 11)

(H) Constitutive heterochromatin.

(*) Centromere.

(p) Short arm. (q) Long arm.

(prox) Proximal.

(inters) Interstitial.

(dist) Distal.

The absence of interstitial telomeric sequences (ITS; Fig 4B and 4D) may indicate that the rearrangements are old and that such sequences may have degenerated to the point of being undetectable by FISH [51, 52]. Alternatively, the rearrangements may have occurred without involving telomeric sequences. Similar results are described for five karyotypes of three *Neacomys* species [28].

Pereira et al. [23] have made a comparative analysis of *Akodon* sp. (ASP, Akodontini, 2n = 10/FN = 14) and *Necromys lasiurus* (NLA, Akodontini, 2n = 34/FN = 34) with *Cerradomys langguthi* (CLA, Oryzomyini, 2n = 46/FN = 62) [20], *Thaptomys nigrita* (TNI, Akodontini, 2n = 52/FN = 52) and *Akodon montensis* (AMO, Akodontini, 2n = 24/FN = 42) [22], all hybridized with HME probes. These results highlight some exclusive characters from the Akodontini tribe and some ancestral traits for the Sigmodontinae subfamily.

**Fig 9:**
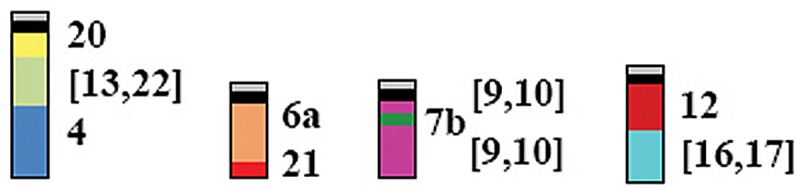
Possible synapomorphic characters of *Neacomys* genus.

When we compare those authors’ results with the NSP-A and NSP-B (Oryzomyini) karyotypes (Table 2), and extrapolating them using G-banding for the five karyotypes of three *Neacomys* species (*Neacomys* sp., *N*. *dubosti* and *N*. *paracou*) [28], we observe that the associations HME 20/[13,22], 6/21 and 7/[9,10], which are ancestral traits for Sigmodontinae according to Pereira et al. [23], are present also in *Neacomys*. However, these segments are rearranged in the genus and so they are synapomorphies: the first was due to a fusion with HME 4, generating HME 20/[13,22]/4; the second was due to a fission in the segment that corresponds to HME 6, generating HME 6a/21 and 6b; the third was due to a fission in the HME 7 segment, followed by a paracentric inversion, generating HME [9,10]/7b/[9,10] (Fig 9).

Although HME 1/12 and 5/[16,17] associations may be considered ancestral traits for the Sigmodontinae [23], they are absent in *Neacomys*. The association *HME 12/[16,17] is present in *Neacomys* and is considered to be a chromosomal signature for the genus. We assume that this segment originated from a fission of HME 5/[16,17], followed by a fusion with HME [16,17] and 1/12 segments, generating HME 1/*/12/[16,17] (NSP-B 4, Fig 3); in the other species another fission occurred, generating the synapomorphy of the genus HME 12/[16,17] (Fig 9).

The association HME 19/14/19 is absent in NSP-B, but present as a derived form in NSP-A 25 (HME 19/14/*/23), with only a small segment of HME 19 in NSP-A, while the bigger fragment of HME 19 (NSP-A 14) is not associated. HME 26 is a symplesiomorphic character in NSP-B 17, while NSP-A 27 (HME [13,22]/*/26) is a derivative form. The association HME 11/[16,17] is absent in NSP-A and NSP-B.

In our comparative analysis (Table 2), we observed another trait that could belong to the hypothetical ancestral karyotype of the Sigmodontinae subfamily: HME 15 non-associated, being a symplesiomorphy in TNI 19, CLA 12, NSP-A 15 and NSP-B 7. We consider that the acrocentric HME 24 is the ancestral form, being a symplesiomorphy in TNI 14, CLA 14, NSP-A 11 and NSP-B 11, and that the metacentric form (HME) and associated (AMO 6, ASP 3, NLA 9) are derivative. We also propose other ancestral traits for the Oryzomyini tribe: HME 8 disassociated in two fragments, HME 18 non-associated and HME 25 non-associated.

### Biogeography in *Neacomys*

The geographical barrier of the Amazonian rivers [6] explains the lack of gene flow between interpluvial regions in Amazon, and confines some species to a single endemic area [5], as described for several groups of terrestrial vertebrates, including primates [53], birds [54] and rodents [55]. In the *Neacomys* genus, some species occur in more than one endemic area [2, 24–28], which is in disagreement with the pattern observed for the three undescribed species of *Neacomys*, who have isolated distributions: *Neacomys* sp. within the Marajó island and Xingu endemic area [28], *Neacomys* sp. A within the Tapajós endemic area, and *Neacomys* sp. B within the Rondônia endemic area.

In contrast to the low node support observed for *Neacomys* sp. B, our divergence time estimates (Fig 7) recovered with high statistical support indicates that the geographical area of the ancestor of *Neacomys* sp. + *Neacomys* sp. A was the current Xingu and Tapajós areas of endemism and Marajó Island, while the *Neacomys* sp. ancestor area was the current endemic area of Xingu and Marajó Island.

Our divergence time estimates (Fig 7) suggest that the diversification of *Neacomys* sp. B and *Neacomys* sp. A + *Neacomys* sp. occurred about 0.74 Ma, and the last split was between *Neacomys* sp. A and *Neacomys* sp. about 0.45 Ma. Based on speciation events in genus *Psophia* (Aves) and not on geological analyses, Ribas et al. [56] proposed that the Tapajós river drainage system was developed approximately 1.3–0.8 Ma, whereas Tocantins and Xingu rivers drainage systems were established about 0.8–0.3 Ma, acting as isolating barriers and creating the Tapajós, Xingu and Belém endemic areas.

Those divergence time estimates and diversification are within the range and in agreement with the gradient of chromosomal and molecular differentiation (see *Karyotypic and phylogenetic analyses of Neacomys* and *Chromosomal Rearrangements and Signatures*), which shows that *Neacomys* sp. [28], *Neacomys* sp. A and *Neacomys* sp. B form a monophyletic group, while the first two are sister species (Figs 5 and 6) and share more chromosomal similarities with each other than with *Neacomys* sp. B, that presents derivative chromosomal forms.

Therefore, our data supports the hypothesis that the common ancestor from these taxa was distributed through the eastern Amazon and the Tapajós and Xingu rivers formation and also Marajó Island separation of the continent act as isolating barriers to gene flow and determine the pattern of diversification of these three undescribed species. Thus, our data provide strong support for the Riverine Barrier Hypothesis [7–9].

We emphasize that NSP-A and NSP-B were collected in areas not yet related to any other previously described species or distribution areas corresponding to them, thus enlarging the geographic distribution of the *Neacomys* genus [2, 24–28], for the southwestern region of the Pará state (Brazil). The number of species within the genus and their geographical boundaries remain uncertain [2].

## Conclusions

The comparative chromosomal and molecular analyses in this study demonstrate that the Xingu and Tapajós Rivers act as geographic barriers for these three undescribed *Neacomys* species, delimiting *Neacomys* sp. distribution within the Marajó Island and Xingu endemic areas, NSP-A within the Tapajós endemic area and NSP-B within the Rondônia endemic area. In addition, we establish four synapomorphies for *Neacomys* (associations HME 20/[13,22]/4, 6a/21, [9,10]/7b/[9,10] and 12/[16,17]) and ancestral traits for the Oryzomyini tribe (HME 8a and 8b, 18 and 25) and Sigmodontinae subfamily (HME 15 and 24). It is important to continue using HME probes as taxonomic markers in other Sigmodontinae, for the definition of each tribe’s chromosomal signatures and for the elucidation of taxonomic and phylogenetic relationships.

## Supporting information

S1 Fig Hybridization of each HME whole chromosome probe on *Neacomys* species. A) *Neacomys* sp. A (2n = 58/FN = 68). B) *Neacomys* sp. B (2n = 54/FN = 66). The numbers on white circle refer to HME pair number.
(TIF)

S1 Table List of sequenced specimens included in the molecular analysis of Cytochrome b (*Cyt*b) and Cytochrome C Oxidase—Subunit I (COI) in the present study. For each species the museum number or museum acronym, GenBank accession number and collecting locality are provided. Brazilian (BR) states are Amazonas (AM), Acre (AC) and Pará (PA). CO (Colombia), EC (Ecuador), GN (Guyana), PE (Peru), SR (Suriname) and VE (Venezuela). (*) Sequences gently provided by J. L. Patton. (**) Karyotyped specimens in this study. The numbers in parentheses refer to the localities shown in Fig 1. References are: 1. Catzeflis & Tilak (2009); 2. iBOL (2011); 3. Patton et al. (2000); 4. Hanson & Bradley (2008); 5. Borisenko et al. (2008); 6. da Silva et al. (2015); 7. Miranda et al. (2008); 8. iBOL (2012).
(DOCX)
