# Supplementary figures and images for: Chromosomal diversity and molecular divergence among three undescribed species of Neacomys (Rodentia, Sigmodontinae) separated by Amazonian rivers
Source: PLoS One. 2017 Aug 1;12(8):e0182218. doi: 10.1371/journal.pone.0182218 (PMC5538659; doi:10.1371/journal.pone.0182218)

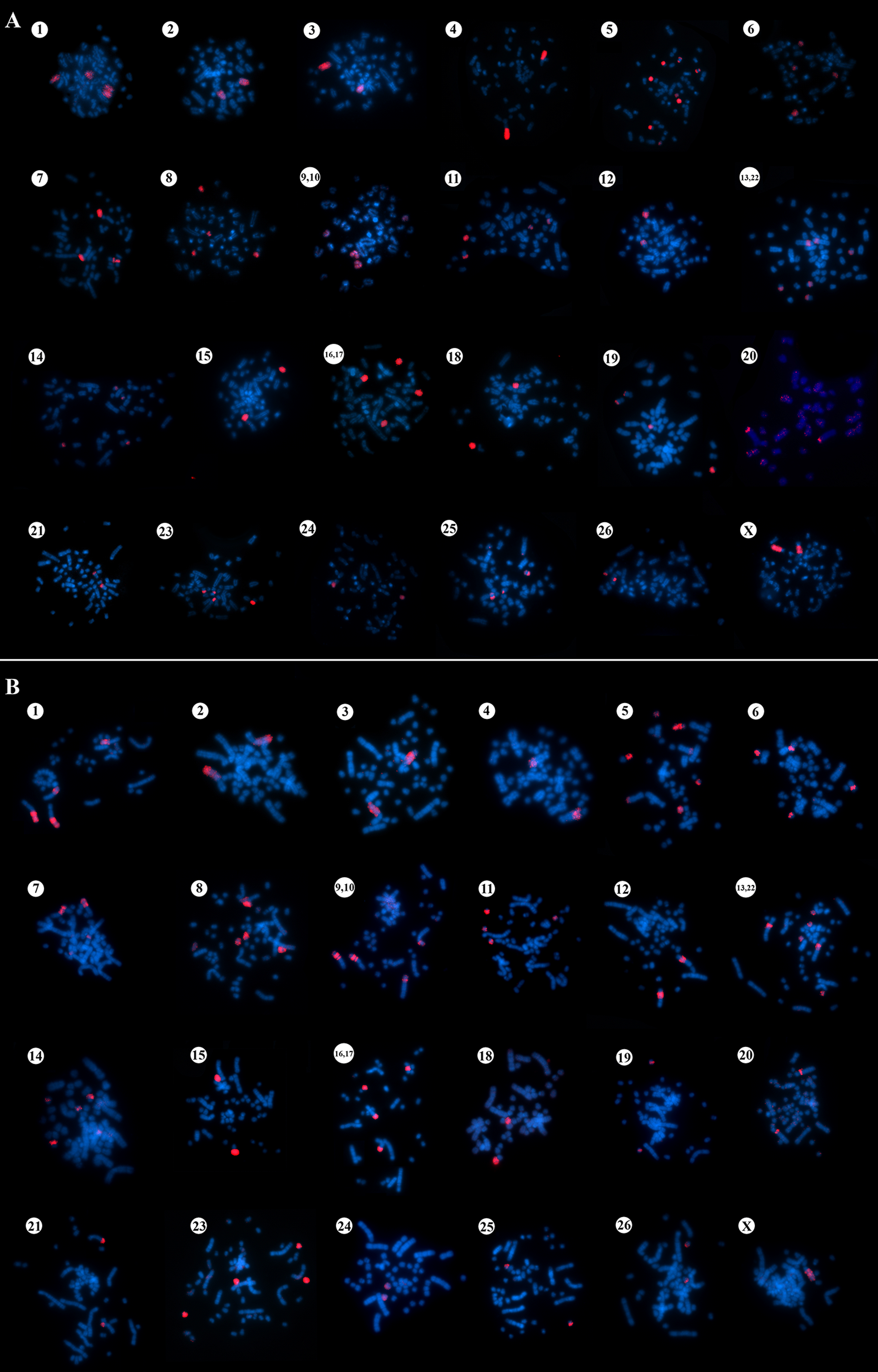

Supplement: S1 Fig — A) Neacomys sp. A (2n = 58/FN = 68). B) Neacomys sp. B (2n = 54/FN = 66). The numbers on white circle refer to HME pair number. (TIF) [file pone.0182218.s001.tif]
