# Supplementary material for: Chromosomal diversity and molecular divergence among three undescribed species of Neacomys (Rodentia, Sigmodontinae) separated by Amazonian rivers
Source: PLoS One. 2017 Aug 1;12(8):e0182218. doi: 10.1371/journal.pone.0182218 (PMC5538659; doi:10.1371/journal.pone.0182218)
Supplement: S1 Table — For each species the museum number or museum acronym, GenBank accession number and collecting locality are provided. Brazilian (BR) states are Amazonas (AM), Acre (AC) and Pará (PA). CO (Colombia), EC (Ecuador), GN (Guyana), PE (Peru), SR (Suriname) and VE (Venezuela). (*) Sequences gently provided by J. L. Patton. (**) Karyotyped specimens in this study. The numbers in parentheses refer to the localities shown in Fig 1. References are: 1. Catzeflis & Tilak (2009); 2. iBOL (2011); 3. Patton et al. (2000); 4. Hanson & Bradley (2008); 5. Borisenko et al. (2008); 6. da Silva et al. (2015); 7. Miranda et al. (2008); 8. iBOL (2012). (DOCX) [file pone.0182218.s002.docx]

**Supplementary Table 1.** **List of sequenced specimens included in the molecular analysis of Cytochrome b (*Cyt*b) and Cytochrome C Oxidase – Subunit I (COI) in the present study.** For each species the museum number or museum acronym, GenBank accession number and collecting locality are provided. Brazilian (BR) states are Amazonas (AM), Acre (AC) and Pará (PA). CO (Colombia), EC (Ecuador), GN (Guyana), PE (Peru), SR (Suriname) and VE (Venezuela). (*) Sequences gently provided by J. L. Patton . (**) Karyotyped specimens in this study. The numbers in parentheses refer to the localities shown in Figure 1. References are: 1. Catzeflis & Tilak (2009); 2. iBOL (2011); 3. Patton et al. (2000); 4. Hanson & Bradley (2008); 5. Borisenko et al. (2008); 6. da Silva et al. (2015); 7. Miranda et al. (2008); 8. iBOL (2012).

| **Species** | **Field number** | **Museum number** | **Voucher** | **CYTB** | **COI** | **GenBank Code** | **Locality** | **Reference** |
| --- | --- | --- | --- | --- | --- | --- | --- | --- |
| *N. dubosti* |  | CM76846 | FM210781 | X |  | FM210781 | SR: Marowijni District, Oelemarie | 1 |
| *N. dubosti* |  |  | ROMMAM 120288 |  | X | HQ919651 | SR | 2 |
| *N. guianae* |  | CM76847 | FM210778 | X |  | FM210778 | SR: Marowijni District, Oelemarie | 1 |
| *N. guianae* |  |  | ROM 106708 |  | X | JF491470 | GN: Upper Takutu-Upper Essequibo | 2 |
| *N. minutus* | MNFS624 | INPA3891 | ***** | X |  |  | BR: AM, Sacado, right bank Rio Juruá | 3 |
| *N. musseri* | MNFS1395 | INPA3046 | ***** | X |  |  | BR: AC, Igarapé Porongaba, right bank Rio Juruá | 3 |
| *N. paracou* | F41218 | ROM114143 | FM210766 | X |  | FM210766 | SR: Brownsberg, Nature Park | 1 |
| *N. paracou* |  |  | ROM 108874 |  | X | JF491472 | GN: Potaro-Siparuni, Gorge Camp, 40 Km SSW, of Kurupukari, Iwokrama Reserve. | 2 |
| *N. spinosus* | JLP7627 | MVZ155015 | ***** | X |  |  | PE: AM, Huampami, Río Cenepa | 3 |
| *N. spinosus* |  |  | ROM 106105 |  | X | EU095446 | EC: Napo | 5 |
| *Neacomys* sp. (2) | MAJ41 | MPEG40443 |  | X |  | KX752072 | BR: PA, Chaves. | 6 |
| *Neacomys* sp. (2) | MAJ49 | MPEG40440 |  | X |  | KX752075 | BR: PA, Chaves. | 6 |
| *Neacomys* sp. (2) | MAJ35 | MPEG40439 |  | X |  | KX752074 | BR: PA, Chaves. | 6 |
| *Neacomys* sp. (2) | MAJ33 | MPEG40446 |  | X |  | KX752080 | BR: PA, Chaves. | 6 |
| *Neacomys* sp. (1) | PSA184 | MPEG41805 |  | X |  | KX752076 | BR: PA, Marabá, Salobo. | 6 |
| *Neacomys* sp. (1) | PSA79 | MPEG41804 |  | X |  | KX752073 | BR: PA, Marabá, Salobo. | 6 |
| *Neacomys* sp. A (7) | JH04 | UFPAM 1467 |  |  | X |  | BR: PA, Tapajós River Left Bank, Mamãe-Anã community, Jacareacanga municipality (05°47'38.06"S 57°23'57.90"W). | Present Work |
| *Neacomys* sp. A** (8) | JL 14 | UFPAM 1530 |  |  | X |  | BR: PA, Tapajós River Left Bank, Mangabal community, Itaituba municipality (05º22'31.60"S 56º55'25.60"W). | Present Work |
| *Neacomys* sp. A (7) | JH 62 | UFPAM 1669 |  |  | X |  | BR: PA, Tapajós River Left Bank, Mamãe-Anã community, Jacareacanga municipality ( 05°47'38.06"S 57°23'57.90"W). | Present Work |
| *Neacomys* sp. A (7) | JMIH_01 | UFPAM 1277 |  | X | X |  | BR: PA, Tapajós River Left Bank, Mamãe-Anã community, Jacareacanga municipality ( 05°47'38.06"S 57°23'57.90"W). | Present Work |
| *Neacomys* sp. A (7) | JMIH_10 | UFPAM 1284 |  | X | X |  | BR: PA, Tapajós River Left Bank, Mamãe-Anã community, Jacareacanga municipality ( 05°47'38.06"S 57°23'57.90"W). | Present Work |
| *Neacomys* sp. A (6) | JF005 | UFPAM 1444 |  | X | X |  | BR: PA, Tapajós River Left Bank, Itaituba municipality (05°40'0.35"S 57°14'23.13"W). | Present Work |
| *Neacomys* sp. A (7) | JH02 | UFPAM 1465 |  | X | X |  | BR: PA, Tapajós River Left Bank, Mamãe-Anã community, Jacareacanga municipality ( 05°47'38.06"S 57°23'57.90"W). | Present Work |
| *Neacomys* sp. A (7) | JH27 | UFPAM 1487 |  | X | X |  | BR: PA, Tapajós River Left Bank, Mamãe-Anã community, Jacareacanga municipality ( 05°47'38.06"S 57°23'57.90"W). | Present Work |
| *Neacomys* sp. A** (8) | JL 03 | UFPAM 1520 |  | X | X |  | BR: PA, Tapajós River Left Bank, Mangabal community, Itaituba municipality (05º22'31.60"S 56º55'25.60"W). | Present Work |
| *Neacomys* sp. A** (5) | JB025 | UFPAM 1577 |  | X | X |  | BR: PA, Tapajós River Left Bank, Mangabal community, Itaituba municipality (05º13'36.90"S 56º55'46.6"W). | Present Work |
| *Neacomys* sp. A** (5) | JB031 | UFPAM 1583 |  | X | X |  | BR: PA, Tapajós River Left Bank, Mangabal community, Itaituba municipality (05º13'36.90"S 56º55'46.6"W). | Present Work |
| *Neacomys* sp. A (7) | JH 40 | UFPAM 1647 |  | X | X |  | BR: PA, Tapajós River Left Bank, Mamãe-Anã community, Jacareacanga municipality ( 05°47'38.06"S 57°23'57.90"W). | Present Work |
| *Neacomys* sp. A (7) | JH 47 | UFPAM 1654 |  | X | X |  | BR: PA, Tapajós River Left Bank, Mamãe-Anã community, Jacareacanga municipality ( 05°47'38.06"S 57°23'57.90"W). | Present Work |
| *Neacomys* sp. A (9) | JJ 09 | UFPAM 1691 |  | X | X |  | BR: PA, Tapajós River Left Bank, São Martins community, Jacareacanga municipality (06° 6'39.14"S 57°36'19.88"W). | Present Work |
| *Neacomys* sp. B** (4) | JMIC_06 | UFPAM 1227 |  |  | X |  | BR: PA, Tapajós River Right Bank, Penedo community, Itaituba municipality (05º27'12.7"S 57º05'6.3"W). | Present Work |
| *Neacomys* sp. B** (3) | LTJ 12 | UFPAM 1995 |  | X | X |  | BR: PA, Tapajós River Right Bank, Juruti minicipality (02°18'12.8"S 56°06'03.4"W). | Present Work |
| *Neacomys* sp. B (3) | LTJ 21 | UFPAM 2002 |  | X | X |  | BR: PA, Tapajós River Right Bank, Juruti minicipality (02°18'12.8"S 56°06'03.4"W). | Present Work |
| *Neacomys* sp. B (3) | LTJ 22 | UFPAM 2003 |  | X |  |  | BR: PA, Tapajós River Right Bank, Juruti minicipality (02°18'12.8"S 56°06'03.4"W). | Present Work |
| *Neacomys* sp. B** (3) | LTJ 23 | UFPAM 2004 |  | X | X |  | BR: PA, Tapajós River Right Bank, Juruti minicipality (02°18'12.8"S 56°06'03.4"W). | Present Work |
| *Neacomys* sp. B** (3) | LTJ 36 | UFPAM 2015 |  | X | X |  | BR: PA, Tapajós River Right Bank, Juruti minicipality (02°18'12.8"S 56°06'03.4"W). | Present Work |
| *Neacomys* sp. B** (3) | LTJ 42 | UFPAM 2019 |  | X | X |  | BR: PA, Tapajós River Right Bank, Juruti minicipality (02°18'12.8"S 56°06'03.4"W). | Present Work |
| *H. megacephalus* |  | MHNLS8061 | EU579499 | X |  |  | VE: Bolivar, San Ignacio de Yuruani | 4 |
| *H. megacephalus* |  |  | ROM:MAM 120619 |  | X |  | SR: Sipaliwini River Camp | 8 |
| *Oecomys concolor* |  |  | MN36350 | X |  |  | No information available | 7 |
| *Oecomys rutilus* |  |  | ROM 104473 |  | X |  | EC: Napo | 5 |

1. Catzeflis F, Tilak M: Molecular systematic of Neotropical spiny mice (*Neacomys*: Sigmodontinae, Rodentia) from the Guiana Region. Mammalia. 73: 239- 247 (2009).
2. International Barcode of Life (iBOL). Direct Submission. Submitted (24-JAN-2011). Biodiversity Institute of Ontario, University of Guelph, 50 Stone Rd West, Guelph, Ontario N1G2W1, Canada;
3. Patton JL, Silva MNF, Malcolm JR: Mammals of the Rio Juruá and the Evolutionary and ecological diversification of Amazonia. Bulletin of the American Museum of Natural History. 244: 202-292 (2000).
4. Hanson JD, Bradley RD: Molecular phylogenetics of Oryzomyini: does a multi-gene approach help resolve a systematic conundrum? Unpublished. Submitted (06-MAR-2008). Biological Sciences, Texas Tech University, 43131, Lubbock, TX 79409, USA.
5. Borisenko AV, Lim BK, Ivanova NV, Hanner RH, and Hebert PDN. 2008. DNA barcoding in surveys of small mammal communities: a field study in Suriname. Molecular Ecology Notes, 8: 471-479.  (2008).
6. Da Silva WO, Pieczarka JC, Rossi RV, Schneider H, Sampaio I., Miranda CL, da Silva CR, Cardoso EM, Nagamachi CY. Diversity and karyotypic evolution in the genus Neacomys (Rodentia, Sigmodontinae). Cytogenetic and Genome Research. 2015; 146: 296-305.
7. [Miranda GB](http://www.uniprot.org/uniprot/?query=author:%22Miranda+G.B.%22), [Andrades-Miranda J](http://www.uniprot.org/uniprot/?query=author:%22Andrades-Miranda+J.%22), [Oliveira LFB](http://www.uniprot.org/uniprot/?query=author:%22Oliveira+L.F.B.%22), [Langguth A](http://www.uniprot.org/uniprot/?query=author:%22Langguth+A.%22), [Mattevi MS.](http://www.uniprot.org/uniprot/?query=author:%22Mattevi+M.S.%22) Species relationships and geographic patterns of genetic variation in Oecomys genus (Rodentia, Sigmodontinae) based on sequences of cyt-b and RAG2 genes. Submitted (05-OCT-2008) GGP Genetica e Toxicologia Aplicada, Universidade Luterana do Brasil (ULBRA), Canoas, RS 92420-280, Brazil.
8. International Barcode of Life (iBOL). Direct Submission. Submitted (28-JAN-2012). Biodiversity Institute of Ontario, University of Guelph, 50 Stone Rd West, Guelph, Ontario N1G2W1, Canada.
